# MMG-Based Motion Segmentation and Recognition of Upper Limb Rehabilitation Using the YOLOv5s-SE

**DOI:** 10.3390/s25072257

**Published:** 2025-04-03

**Authors:** Gangsheng Cao, Shen Jia, Qing Wu, Chunming Xia

**Affiliations:** 1 School of Mechanical and Power Engineering, East China University of Science and Technology, Shanghai 200237, China; gangshengcao@mail.ecust.edu.cn (G.C.); 22012965@mail.ecust.edu.cn (S.J.); 2 School of Mechanical and Automotive Engineering, Shanghai University of Engineering Science, Shanghai 201620, China

**Keywords:** mechanomyography, upper limb motion segmentation, pattern recognition, target detection, YOLOv5s-SE, deep learning

## Abstract

Mechanomyography (MMG) is a non-invasive technique for assessing muscle activity by measuring mechanical signals, offering high sensitivity and real-time monitoring capabilities, and it has many applications in rehabilitation training. Traditional MMG-based motion recognition relies on feature extraction and classifier training, which require segmenting continuous actions, leading to challenges in real-time performance and segmentation accuracy. Therefore, this paper proposes an innovative method for the real-time segmentation and classification of upper limb rehabilitation actions based on the You Only Look Once (YOLO) algorithm, integrating the Squeeze-and-Excitation (SE) attention mechanism to enhance the model’s performance. In this paper, the collected MMG signals were transformed into one-dimensional time-series images. After image processing, the training set and test set were divided for the training and testing of the YOLOv5s-SE model. The results demonstrated that the proposed model effectively segmented isolated and continuous MMG motions while simultaneously performing real-time motion category prediction and outputting results. In segmentation tasks, the base YOLOv5s model achieved 97.9% precision and 98.0% recall, while the improved YOLOv5s-SE model increased precision to 98.7% (+0.8%) and recall to 98.3% (+0.3%). Additionally, the model demonstrated exceptional accuracy in predicting motion categories, achieving an accuracy of 98.9%. This method realizes the automatic segmentation of time-domain motions, avoids the limitations of manual parameter adjustment in traditional methods, and simultaneously enhances the real-time performance of MMG motion recognition through image processing, providing an effective solution for motion analysis in wearable devices.

## 1. Introduction

Upper limb movement plays a crucial role in people’s daily lives and work. Currently, there are approximately 15 million stroke patients worldwide [1]. Due to the high disability rate of stroke, many patients present hemiplegia symptoms even after surgery, resulting in the inability of these patients to achieve normal upper limb movement, which seriously affects their daily lives [2]. To facilitate the recovery of stroke patients, targeted rehabilitation training methods, including passive training, should be promptly implemented to elicit active responses in the hemiplegic limbs and restore their normal functional capabilities [3]. For hemiplegic patients, mirror training is an effective rehabilitation training strategy. With the advancement of technology, mirror training therapy assisted by exoskeletons has gradually emerged [4]. The primary mechanism of this approach involves the patient’s healthy side performing rehabilitation movements, which subsequently drives the hemiplegic side to execute symmetrical motions with the assistance of exoskeletons and robots. The mirror movement strategy not only facilitates the restoration of limb function but also provides continuous brain stimulation, thereby promoting the remodeling of neural pathways between the limbs and the brain while maintaining limb symmetry and coordination, offering distinct advantages [5]. For the effective execution of exoskeleton-assisted movements under this strategy, accurate and rapid recognition of the healthy upper limb’s motions is essential.

As technology has advanced, more and more technical approaches have been applied to motion recognition, particularly the integration of machine learning techniques into the field of motion pattern recognition, which has expanded the application directions of rehabilitation training. Currently, the following three data types are applied to machine learning-based motor pattern recognition for upper limb rehabilitation training: skeletal data [6], image data [7], and biomedical signals [8]. Skeletal data is usually acquired through motion capture devices or depth cameras, such as Kinect. This type of data directly reflects the position and movement trajectory of joints but is limited by the equipment and can only be used in specific environments, making it unsuitable for home-based rehabilitation. Image data can capture rich visual information but demands significant computational resources and is susceptible to factors such as lighting and occlusion. In contrast, biomedical signals offer unique advantages in reflecting subjective motion intentions and follow-through, while also providing real-time feedback on physiological activities, thus being widely applied in upper limb rehabilitation training. With the rapid development of sensor technology, surface electromyography (sEMG) [9] and MMG [10] are widely used in upper limb movement pattern recognition. sEMG measures the surface potential changes of muscles, recording the electrical signal fluctuations generated during muscle fiber contraction, and sEMG-based upper extremity movement recognition research has made great progress and applications [11]. However, sEMG is susceptible to electrical noise, motion artifacts, and sweating [12], and due to these limitations, MMG is beginning to receive increasing attention from researchers. MMG is a mechanical signal that assesses muscle activity by measuring minute mechanical vibrations or displacements on the muscle surface [10] and can be acquired by accelerometers. It is typically caused by muscle contraction-induced vibrations or fluctuations and can reflect dynamic muscle performance, such as strength, fatigue, and working state. Because MMG is inherently a mechanical signal, it requires less stringent sensor placement as compared with sEMG, does not necessitate skin surface preparation and can even be collected through thin fabric [13]. Additionally, MMG is less susceptible to interference from other electrical signals, ensuring signal integrity and accuracy during acquisition [14]. MMG is widely applied in muscle motion pattern recognition [15], joint acceleration estimation [16], muscle fatigue studies [17], and sports injury prevention [18]. Liu et al. [19] developed a MMG-based novel wearable human–machine interface, extracting features from wavelet packet decomposition time signals and coefficients, and employing sequential forward selection to identify significant features, achieving highly accurate hand motion. Min et al. [17] applied constant electrical stimulation to the tibialis anterior muscle of 21 subjects, determining muscle fatigue levels based on MMG changes and establishing a linear relationship between MMG signals and muscle fatigue. Mateusz et al. [20] designed an MMG sensor composed of two coupled piezoelectric discs, obtaining the relationship between force and MMG under static conditions and classifying gesture movements using a neural network, thereby simplifying the spatial layout of the MMG-based HMI interface. Meagher et al. [21] designed a wearable sensor device based on MMG, integrating inertial measurement units with MMG for post-stroke arm rehabilitation.

MMG-based motion pattern recognition methods usually follow the following standardized process: (1) signal preprocessing and motion segmentation, (2) feature extraction and selection, (3) classifier training, and (4) motion pattern recognition. The motion segmentation process mainly uses segmentation methods such as the energy-based method and the sliding window method [22] to achieve automatic segmentation. Among them, the threshold determination of the energy method relies on empirical values and motion pattern features, and the sliding window method sets the window length based on the action execution time. Although these methods realize the automatic segmentation of motions to a certain extent, they still require more manual intervention, and the segmentation effect mainly relies on subjective assessment, which makes it difficult to ensure the accuracy of automatic segmentation. In addition, in application scenarios with high real-time requirements, the traditional “segmentation-feature extraction-model training-recognition” process will significantly increase the system response time and affect the recognition efficiency. Therefore, in this paper, we hope to explore a method that can be used for real-time automatic segmentation and fast recognition of motions.

In recent years, the YOLO algorithm [23], as an efficient target detection algorithm, has achieved remarkable results in many fields, such as urban road traffic sign detection [24] and high-voltage transmission line insulator fault detection [25]. Although there have been studies applying the YOLO algorithm to upper limb movement pattern recognition [26], its application in MMG-based motion recognition remains unexplored. Considering that the YOLO algorithm has the advantages of real-time prediction of target position, fast processing speed, and support for instance segmentation, this study proposes an innovative motion segmentation and classification method based on the YOLO algorithm. The method automatically segments and classifies MMG images generated by continuous actions through computer vision technology, effectively solving the limitations of traditional methods that require manually setting thresholds or performing manual segmentation. To further improve the model performance, this study also introduces the SE attention mechanism into the model.

This paper aims to propose a real-time target detection model, namely YOLOv5s-SE, that can be used for the segmentation and classification of MMG from upper limb movements. In this research, MMG were collected by eight subjects performing seven types of common upper-limb rehabilitation movements, and some of the signal images were subjected to data processing and annotation to use for the YOLOv5s-SE model training. The experimental results show that the trained YOLOv5s-SE can effectively process continuous MMG signal images and realize automatic segmentation and classification of motions.

The remainder of this paper is organized as follows: Section 2 includes the experimental protocols related to MMG acquisition, experimental data processing, and the specific model structure of YOLOv5s-SE. Section 3 describes various experimental results. Finally, Section 4 provides a discussion of experimental results and summarizes the findings of this paper and the future work.

## 2. Materials and Methods

### 2.1. Experimental Program

**Figure 1:**
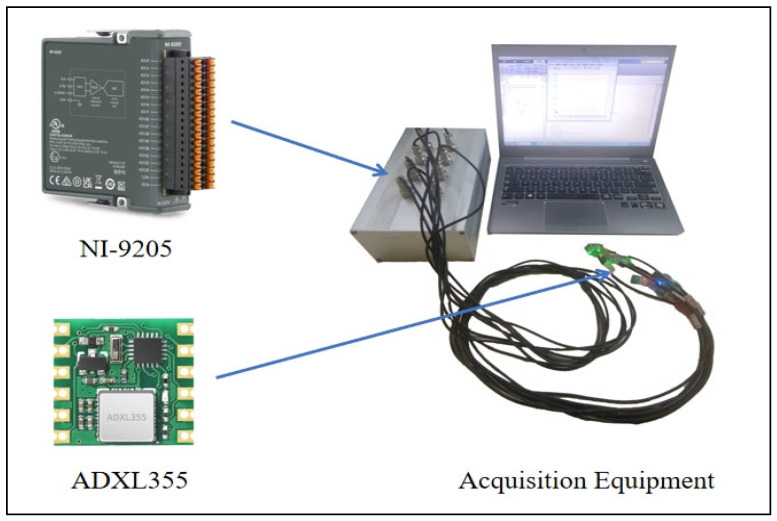
MMG acquisition equipment.

The upper limb MMG signals in this experiment were collected by ADXL355 accelerometer (Analog Devices, Inc., Wilmington, MA, USA), and the acquired MMG signals were also digitized using a 16-bit A/D converter card (NI-9205, Austin, TX, USA) and ultimately obtained as MMG signal images. The acquisition equipment is shown in Figure 1.

The sampling frequency used in the experiment is 1000 Hz, and the experimental data are analyzed and processed in MATLAB 2022b and Python 3.11.3. The relevant models are trained, and model accuracy is verified in this study.

**Figure 2:**
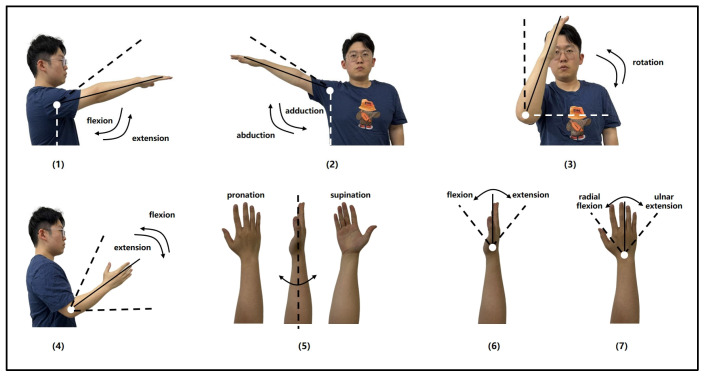
Seven types of upper extremity rehabilitation training movement. (1) Shoulder flexion; (2) Shoulder abduction; (3) Shoulder internal rotation; (4) Elbow flexion; (5) Wrist internal rotation; (6) Wrist flexion; (7) Wrist abduction.

The participants of this experiment were eight males; all of them were in the age range of 23–25 years old, their height was in the range of 170–180 cm, their weight was in the range of 65–75 kg, their right hands were their dominant hands, and all of them were in good health and had no history of neuromuscular disorders. Before the experiment, all participants underwent training to familiarize themselves with the experimental procedures and signed the informed consent form. In order to better simulate the condition of hemiplegic patients during upper-limb rehabilitation training, this experiment chose shoulder flexion, shoulder abduction, shoulder internal rotation, elbow flexion, wrist internal rotation, wrist flexion, and wrist abduction for a total of seven categories of common upper-limb rehabilitation training movements, which involved the three major joints of the upper limb: shoulder, elbow, and wrist. These seven upper-limb rehabilitation movement styles are shown in Figure 2. Participants uniformly used the right upper extremity to complete seven types of movements, each movement was performed in four sets of 50 repetitions, resulting in 200 samples per movement type, with a 3-s interval between consecutive movements. To minimize the impact of muscle fatigue, participants rested for one minute after completing each set before proceeding to the next. When performing the above seven types of movements, the human upper limb is mainly powered by five muscles: deltoid, biceps, triceps, brachioradialis, and ulnar flexor carpi radialis; therefore, during the experiment, the ADXL355 sensors were affixed to the right upper limb of the subject at the locations of these muscles to obtain the MMG signals generated by the five muscles.

### 2.2. Data Processing

**Figure 3:**
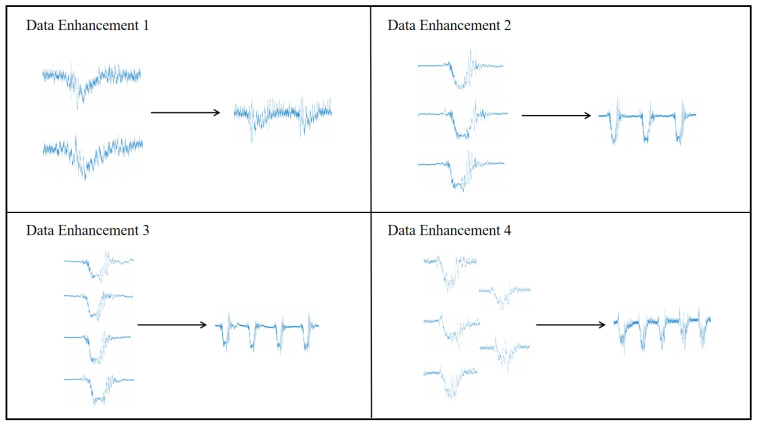
MMG image data enhancement example.

The MMG data collected in this experiment are a one-dimensional time series signal, based on which the corresponding MMG images can be generated. To help the YOLO model to have better segmentation and classification performance, the data in the training set should have obvious features and sufficient quantity. In addition to the original MMG images, this paper also carries out data enhancement processing on some of the collected images of the MMG dataset. The MMG signal images of the same action are stretched and shrunk to a certain degree in the time domain with the vertical scale unchanged, and image blending is performed at the same time to obtain more data and enhance the sample diversity. Examples of the dataset before and after the data enhancement process are shown in Figure 3.

**Figure 4:**
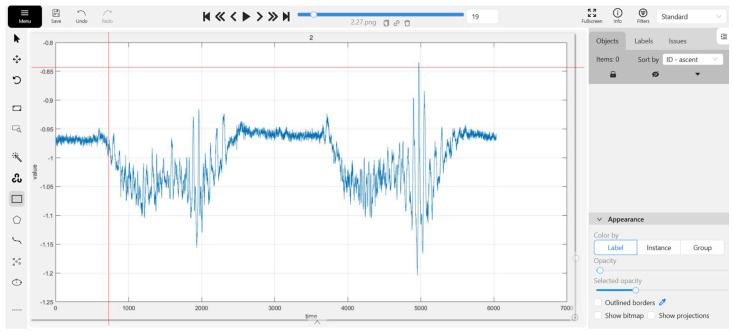
Screenshot of “MMG detection” with CVAT.

After obtaining all MMG images, manual region segmentation was performed on each image using the Computer Vision Annotation Tool (CVAT) website to isolate individual movements. Concurrently, the corresponding movement types were labeled. This process culminated in the establishment of a dataset named “MMG detection”. The interface for manually segmenting and labeling the MMG images is depicted in Figure 4.

The final “MMG detection” consisted of 1912 images, 2552 action signals, and their corresponding labeling information. Among them, 1477 images were selected as the training set, and 435 images were selected as the test set.

### 2.3. YOLOv5-SE Target Detection Model

#### 2.3.1. Network Structure of YOLOv5

The YOLO model has many advantages, including high speed inference, high accuracy, and lightweight design, making it perform well in many computer vision tasks. Its hierarchical module design makes YOLO more flexible and can be adjusted and customized according to task requirements to perform well in different application scenarios. Compared with several YOLO versions released earlier, YOLOv5 has a more concise architecture. It uses the PyTorch framework [27], which simplifies the model implementation and debugging process, makes the training cycle shorter and easier to train and carry on the hardware system, and is suitable for low-power devices (such as embedded devices, mobile devices, etc.) to run to complete the corresponding detection tasks.

The architecture of YOLOv5 includes a backbone network (Backbone), a feature enhancement module (Neck), and an output layer (Head) [28]. The backbone network is based on the CSP Darknet53 structure, and the CSP network is improved based on the traditional residual network (ResNet). By reducing the redundant computation in the model, the training speed is improved, and the diversity of feature representation is increased. The convolution layer of YOLOv5 consists of a convolution kernel and an activation function. By stacking multiple convolution layers, the high-level semantic features of the image are gradually extracted. In addition, YOLOv5 also contains the SPP module and the Focus module. The SPP module processes the image through a variety of pooling operations of different sizes to enhance the network’s ability to identify objects at different scales and the model’s ability to perceive objects at different scales of the image. The Focus module effectively extracts finer-grained spatial information by segmenting the input image and fusing these regions. YOLOv5 provides four models at different scales: YOLOv5s, YOLOv5m, YOLOv5l, and YOLOv5x. YOLOv5s is suitable for scenarios with high-speed requirements and limited resources but with lower accuracy. YOLOv5m provides a balance between speed and accuracy, which is suitable for most applications. YOLOv5l provides higher accuracy and is suitable for tasks requiring high accuracy, but its inference speed is slow. YOLOv5x has the best accuracy and is suitable for high-end applications, but it has the slowest inference speed and high computational resource requirements. The selection of the YOLO model depends on the accuracy requirements and computational resources of the specific task.

**Figure 5:**
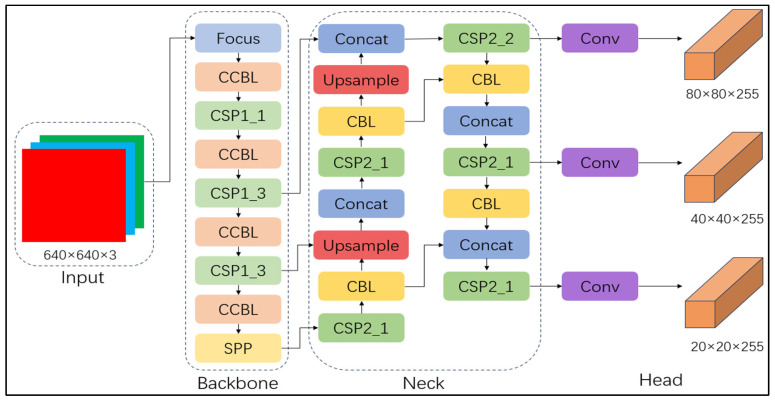
The network architecture of YOLOv5s.

Compared with the other versions, YOLOv5s has the smallest parameter count and memory footprint in the YOLOv5 series. It consists of a weight file equivalent to 27 MB, offering fast inference and low latency; it is suitable for real-time inference or resource-constrained environments, such as embedded systems. Therefore, YOLOv5s was used in this study. The complete network architecture of YOLOv5s is shown in Figure 5.

The primary goal of the YOLO is object detection, which essentially involves instance segmentation of target objects and classification tasks. The predicted bounding box output is expressed as $\left(x,y,w,h,C\right)$, where x and y represent the coordinates of the bounding box’s center, w and h represent its width and height, and C represents the confidence of the predicted result. YOLO predicts bounding boxes for each grid cell and uses formulas to localize, classify, and regress the size of the bounding boxes. The key formulas for the center coordinates x, y, w, and h of the bounding box are as follows:

(1) $$x=\sigma (\hat{x})+c_{x}$$

(2) $$y=\sigma \left(\hat{y}\right)+c_{y}$$

(3) $$w=\exp\left(\hat{w}\right)\times anchor_{w}$$

(4) $$h=\exp\left(\hat{h}\right)\times anchor_{h}$$

where the σ denotes the Sigmoid activation function, $\hat{x}$ and $\hat{y}$ are the predicted offsets relative to the coordinates of the anchor box, $c_{x}$ and $c_{y}$ are the relative positions of the grid cell, $\hat{w}$ and $\hat{h}$ are the predicted width and height offsets relative to the anchor box, and $anchor_{w}$ and $anchor_{h}$ are the predefined anchor box width and height.

For each predicted bounding box, YOLOv5s first predicts a confidence score indicating the probability of the box containing an object. The formula of the confidence C is:

(5) $$C=S_{Objectness}\times IoU$$

where the $S_{Objectness}$ represents the model’s predicted probability of the bounding box containing an object, and IoU represents the intersection ratio between this box and the true bounding box.

Additionally, for the bounding box, the model predicts probabilities for each specific class. For each class i, the class prediction confidence $P_{i}$ is the probability of the box belonging to class i. Thus, the final confidence calculation formula is:

(6) $$\hat{p_{i}}=\sigma \left(\hat{c}\right)\times \sigma \left(\hat{p_{i}}\right)$$

where $\hat{c}$ represents the object confidence of the bounding box, and $\hat{p_{i}}$ is the class confidence for each class i.

#### 2.3.2. SE Attention Mechanism Module

To enhance the model’s adaptive capability to different feature channels, thereby improving the network’s performance, this study introduces the SE attention mechanism [29] into the YOLOv5s framework, incorporating it into the Backbone. The SE attention mechanism is a lightweight adaptive channel attention module that dynamically adjusts the weights of feature channels, enabling the network to better focus on critical information and improving the efficiency of feature utilization.

**Figure 6:**
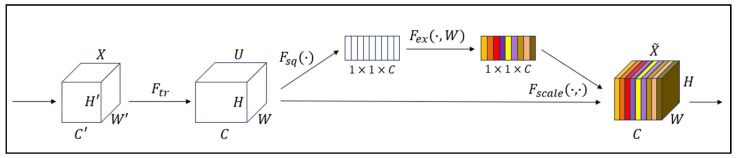
SE attention mechanism algorithm architecture.

The SE attention mechanism can be divided into two operational steps: Squeeze and Excitation. Figure 6 illustrates the algorithmic architecture of the SE attention mechanism.

The input feature map is denoted as X, with height H, width W, and channel number C. The Squeeze operation obtains the global information $Z_{c}$ for each channel by performing global average pooling, where $Z_{c}$ is calculated as follows:

(7) $$Z_{c}=\frac{1}{H\times W}\sum_{i=1}^{H}\sum_{j=1}^{W}x_{c}\left(i,j\right)$$

The Excitation operation processes the global features obtained from the previous step through a fully connected network to generate the excitation weights s for each channel. The calculation of s is as follows:

(8) $$s=F_{ex}\left(z,W\right)=\sigma \left(W_{2}\delta \left(W_{1}z\right)\right)$$

where $W_{1}$ and $W_{2}$ represent the weights of the first and second fully connected layers, and σ denotes the Sigmoid activation function.

To calibrate the output of each channel, the obtained channel weights are applied to each channel of the original feature map $\tilde{X}$ after the excitation operation. The calculation is as follows, where ⊙ denotes element-wise multiplication:

(9) $$\tilde{X}=X⊙s$$

In this study, the MMG signal has complex temporal and nonlinear variation characteristics and is also prone to contain interference noise. Additionally, if SE is added to the model, the model can dynamically weight different frequency bands, time-domain features, or other key signals through the channel attention mechanism to enhance the representation of effective action signals and adaptively reduce the weight of unimportant feature channels, thus effectively suppressing noise and interference and ensuring that the model focuses on more important signal features. Therefore, based on the YOLOv5 model, the SE attention mechanism is introduced in this study with the aim of improving the accuracy and robustness of the model.

### 2.4. Model Performance Evaluation

Precision (P) [30] is a key indicator for evaluating model performance. it quantifies the proportion of true positives (TP) in all positive cases, while false positives (FP) represent regions incorrectly predicted as positive. Recall rate (R) [31] measures the proportion of all actual positive examples that can be correctly detected by the model. It is used to detect whether the model has missed the true target, and the false negative (FN) represents the case when the actual target has not been detected. In the context of detecting MMG, a high recall rate means that the probability of the model missing the action signal is small, so the recall rate is also of high reference value in this study. P and R are calculated as follows:

(10) $$P=\frac{TP}{TP+FP}$$

(11) $$R=\frac{TP}{TP+FN}$$

Average Precision (AP) [31] is a metric obtained by calculating the Precision–Recall (PR) curve under different IoU thresholds by calculating the area under the curve. AP reflects precision performance at various recall rates. In this study, AP was used to measure the combined ability of the model in detecting various types of action signals. The formula for AP is as follows:

(12) $$AP=\int_{0}^{1}P\left(R\right)d\left(R\right)$$

The mean Average Precision (mAP) [31] is obtained by performing a weighted average operation of the AP of all categories, which also provides a comprehensive assessment of the model’s ability to detect under different categories. The formula for mAP is:

(13) $$mAP=\frac{1}{N}\sum_{i=1}^{N}{AP}_{i}$$

During the model training process in this study, these metrics were used to evaluate the model, and corresponding curves were plotted. This approach provided an intuitive representation of the model’s iterative progress during training, and assessed the quality of the model after training.

## 3. Results

### 3.1. Motion Segmentation Image

**Figure 7:**
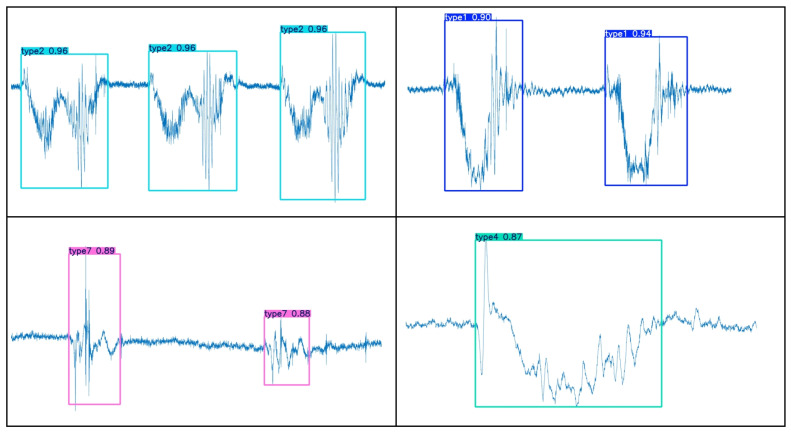
Motion segmentation with YOLOv5s-SE.

**Figure 8:**
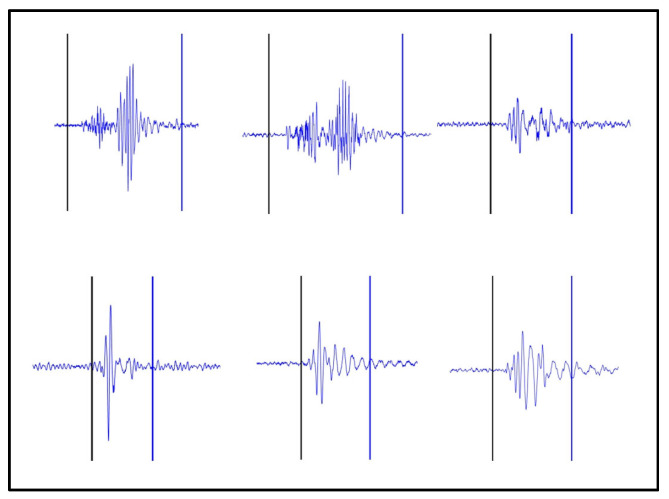
Motion segmentation with the energy method. The black vertical line represents the beginning of the motion and the blue vertical line represents the end of the motion.

YOLOv5s-SE is based on the traditional YOLO target detection framework and realizes the task of segmenting time-domain continuous action signals by predicting the bounding box of each action signal. The specific segmentation effect is shown in Figure 7. To make a visual comparison of the segmentation effect, the energy method is used for motion segmentation, as shown in Figure 8.

By comparing Figure 7 and Figure 8, it can be observed that the YOLOv5s-SE model effectively segments MMG images of different actions and accurately determines the starting points of actions, thereby maximizing the extraction of useful data from action signals. In addition to predicting the bounding boxes (starting points and energy amplitudes) for each action, the model also outputs the corresponding class labels and confidence scores for different actions, as well as the segmentation masks of the objects. These masks describe the actual pixel regions occupied by the objects in the images, and the area of these pixel regions can be utilized for future machine-learning feature extraction.

**Figure 9:**
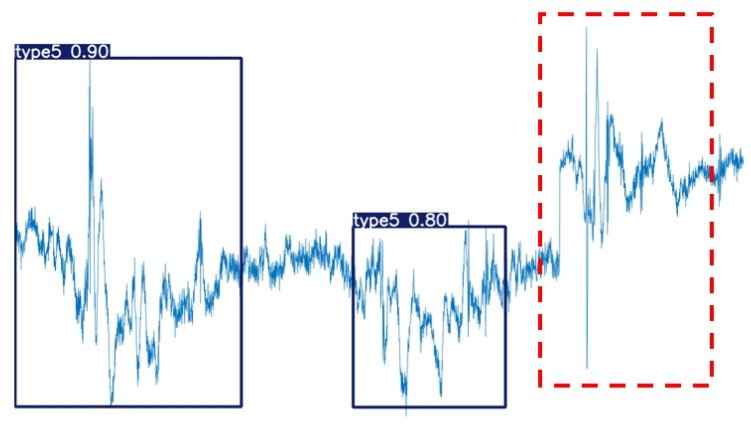
Unrecognized actions without segmentation.

Certainly, due to factors such as the instability of the collected MMG signal strength, errors in the collection device, and limitations in model accuracy, some instances of misidentification and mis-segmentation are inevitable. As shown in Figure 9, the red dashed box contains an action that was neither identified nor segmented because the interval between the two actions was too short.

**Figure 10:**
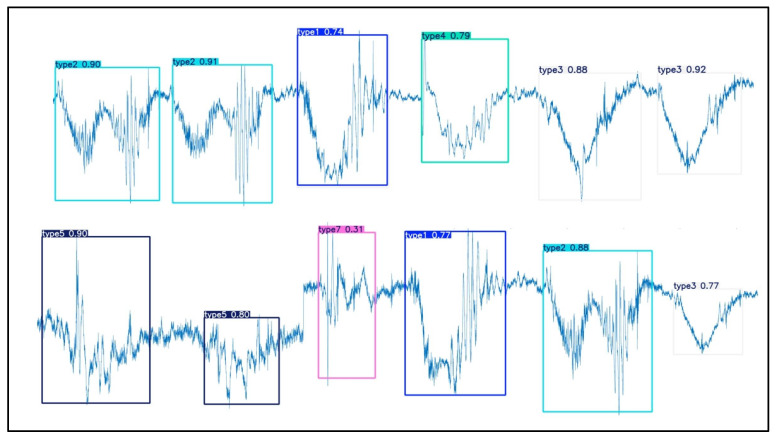
Continuous motion segmentation and recognition result.

Considering the fact that different types of actions may occur randomly during actual rehabilitation training, to simulate this scenario, this study collected multimodal motion-gesture MMG data of various hand movements in the continuous time domain and constructed a validation set. These data were input into a pre-trained model for validation. The validation results, as shown in Figure 10, demonstrate that the model can effectively perform segmentation and recognition tasks even when faced with continuous action data images with a mixture of different kinds of movements.

In conclusion, the classification model can accomplish the segmentation task well for single motion, continuous motions of the same kind, or even continuous motions of different kinds, by segmenting out the corresponding different motions and giving the corresponding labels.

### 3.2. The Model Performance of YOLOv5s and YOLOv5s-SE

The YOLOv5s and YOLOv5s-SE models were trained 100 times each using the test set, and their performance was evaluated across different motion categories as well as overall, as shown in Table 1. The YOLOv5s model achieved an overall precision of 97.9%, a recall of 98.0%, and a mAP of 99.0%. After incorporating the SE attention mechanism, the overall precision increased to 98.7%, the recall rate increased to 98.3%, and the mAP remained at 99.1%. Overall, both models demonstrated excellent performance, with satisfactory results in precision, recall, and mAP, indicating a high degree of overlap between the model predictions and the actual labels in most cases. Relatively speaking, after the incorporation of the SE, the YOLOv5s-SE model exhibited improved precision and recall compared to its pre-enhanced counterpart, demonstrating superior performance.

**Figure 11:**
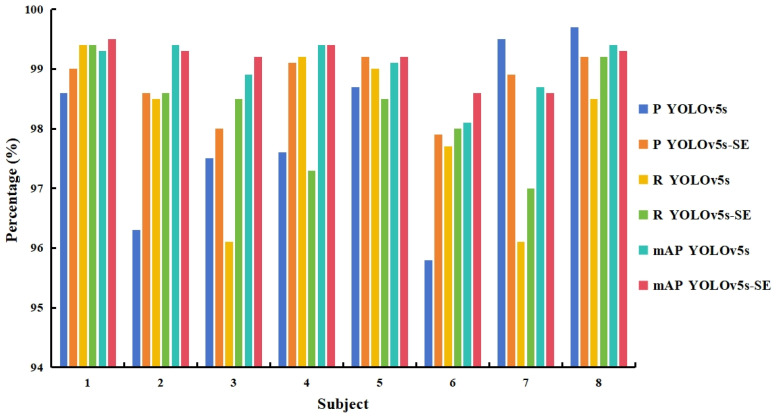
Performance metrics of the two models tested with different subject data.

In this experiment, the MMG signals generated by eight subjects during the execution of upper limb movements were collected and tested for model performance. The model performance metrics obtained from different subjects’ data tests are shown in Figure 11.

In terms of precision, the YOLOv5s model achieved a precision of at least 95.8% for all subjects, and the precision significantly improved after introducing SE, generally reaching above 98%. However, for some subjects, such as Subject 7 and Subject 8, the recognition precision under YOLOv5s was already above 95%, and it slightly decreased after introducing SE. Regarding recall, there was little difference between the two models. For subjects with higher recall rates under YOLOv5s, the recall rate decreased after introducing SE, as observed for Subject 4 and Subject 5. The mAP showed minimal changes, with an overall variation of no more than 1%. Nevertheless, overall, the introduction of SE led to improvements in both precision and recall rates for the model.

**Figure 12:**
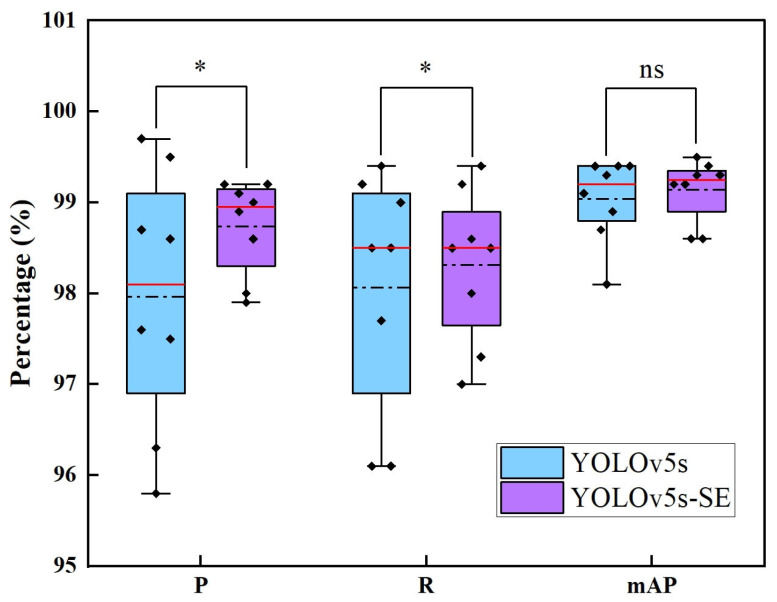
Comparative boxplots of model performance for YOLOv5s and YOLOv5s-SE. the red straight line represents the median value, the black dotted line represents the mean value, the black diamond block represents the specific data, * represents the degree of significant difference and ns represents no significant difference.

In order to better compare and analyze the performance of the two models, the experimental data were statistically analyzed, and a boxplot was produced, and the results are shown in Figure 12 as well as Table 2.

As can be seen from Figure 12, for precision, the median value of YOLOv5s-SE (99.0%) is higher than that of YOLOv5s (98.1%), which indicates that the improved model is better in terms of precision rate as a whole; meanwhile, the interquartile range (IQR) of YOLOv5s-SE is smaller, which indicates that the data distribution is more centralized after the introduction of SE, and the stability has been improved. For recall, the median values of the two models are basically the same, but the box of YOLOv5s-SE is narrower, while YOLOv5s has extreme low values in Subject 3 (96.1%) and Subject 7 (96.1%), and YOLOv5s-SE is close to the lower whisker line only in Subject 4 (97.3%), further confirming the robustness of the enhanced model. For mAP, the two models are essentially close to each other, with highly consistent performance.

As shown in Table 2, significant differences are observed between the two models in terms of precision and recall. Specifically, YOLOv5s-SE demonstrates superior mean values and reduced variances compared with YOLOv5s. The variance of P decreases by 64.3%, while the variance of R is reduced by 48.6%, indicating that the introduction of the SE module significantly enhances the model’s stability. However, in terms of mAP, the performance of the two models is not statistically different. This suggests that the SE module, through its attention mechanism, effectively strengthens critical features, directly improving classification accuracy and model stability while maintaining consistent overall detection performance.

**Figure 13:**
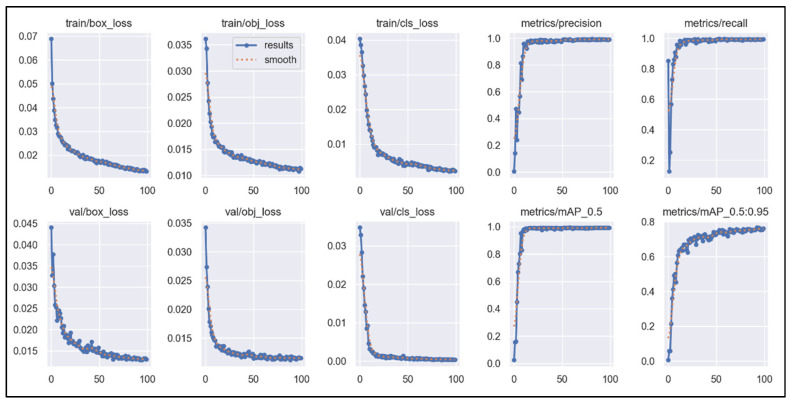
The curve of YOLOv5-SE-related parameters with the number of iterations.

The training results of YOLOv5s evolve as the number of training epochs increases. After introducing the SE attention mechanism, the iterative progression of various parameters during training and validation is depicted in Figure 13. The parameters include the bounding box loss, objectness loss, and classification loss for both the training and validation sets, as well as the model’s precision, recall, mAP, and mAP_0.5:0.95.

Figure 13 shows that as the number of training rounds increases, the values of bounding box regression loss, object loss and category loss gradually decrease. Before reaching approximately 20 epochs, the decreases in these loss values are more pronounced. After 20 epochs, the loss values continue to decrease linearly but at a slower rate. Notably, the objectness loss on the validation set (val/obj_loss) exhibits relatively larger fluctuations after exceeding 20 epochs. For precision, recall, and mAP, these metrics show linear upward trends with increasing epochs, with significant rates of improvement when reaching around 20 epochs. By approximately 20 epochs, these metrics reach a high level and then stabilize, fluctuating within a small range thereafter. For mAP_0.5:0.95, a similar linear upward trend is observed before reaching around 20 epochs, although it does not reach its highest level. After exceeding 20 epochs, mAP_0.5:0.95 continues to show some fluctuations but overall trends upward with a relatively smaller rate of increase.

### 3.3. Motion Pattern Recognition Accuracy Results

**Figure 14:**
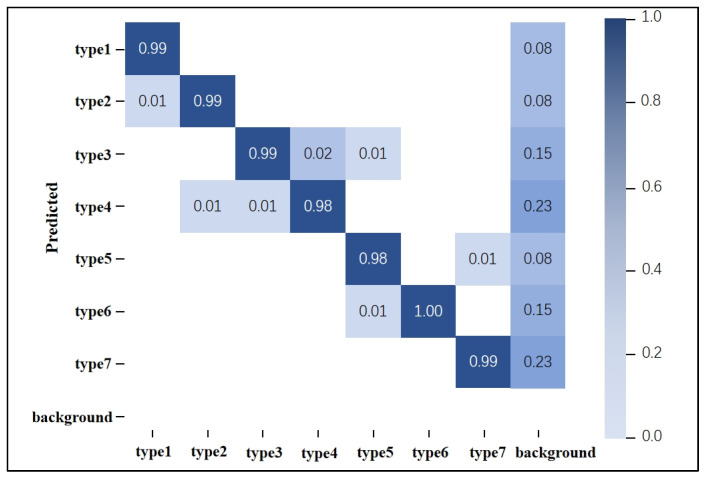
Confusion matrix of motion pattern recognition under YOLOv5-SE.

The YOLOv5s-SE model performs motion segmentation while simultaneously generating corresponding motion labels, effectively recognizing the patterns of actions within the segmented bounding boxes. To evaluate the accuracy of the model’s motion pattern recognition, this study conducted a statistical analysis of the generated motion labels. A portion of the MMG data from eight subjects was used for training, while the rest of the MMG data was used for prediction. The analysis resulted in a motion pattern recognition confusion matrix, as shown in Figure 14. In this matrix, the columns represent the predicted categories by the model, and the rows represent the actual categories. The diagonal values indicate the number of samples correctly predicted by the model, while off-diagonal elements indicate the number of incorrectly predicted samples. Higher values on the diagonal indicate a greater number of correct predictions.

From Figure 14, it can be observed that the pattern recognition accuracy for all actions exceeds 98%, with several actions achieving recognition rates of 99% or even 100%. The average recognition rate is approximately 98.9%. The column labeled “background” represents the probability of predicting an action when the picture is the background (no action signal), and it can be seen that when there is no action signal, the probability of misrecognition by the model is extremely low. The row labeled “background” represents the probability of action signals being misidentified as background. This figure shows that missed detections do not occur. Therefore, the model demonstrates a high level of predictive performance for the seven segmented motion categories, exhibiting excellent upper-limb motion pattern recognition capabilities.

### 3.4. Model Robustness of YOLOv5s

To further investigate the robustness of the model, an additional dataset named MMG detection_2 was created based on the experiment. Compared to the MMG detection dataset used in the experiment, the training set of MMG detection_2 contains hand motion signals from only one subject, while the test set includes hand motion signals from the remaining seven subjects. The same method as in the aforementioned study was employed for training. Ultimately, the overall performance of the YOLOv5s-SE is shown in Table 3.

From Table 3, it can be seen that when the model was trained using MMG from one subject, the trained YOLOv5s-SE model could reach 83.8% precision, 85.8% recall, and 89.8% mAP for the motion recognition of other subjects. This indicates that the model can, to some extent, segment upper-limb rehabilitation training movements while simultaneously performing recognition and classification. Furthermore, by comparing the two trained models, it is evident that the deep model incorporating the SE attention mechanism shows improvements in accuracy, recall, and mAP. However, when compared to the YOLO model trained and tested using MMG signals from the same subject, the accuracy, recall, and other metrics of this model still have room for improvement. There remains significant potential for further research on this issue.

### 3.5. Summary

Based on the experimental results above, the following conclusions can be drawn: For MMG signal processing, the YOLO method effectively accomplishes the dual tasks of action signal classification and continuous time-domain segmentation. By introducing the SE (Squeeze-and-Excitation) attention mechanism into the YOLOv5s architecture and comparing its performance with the original model, the results demonstrate that the YOLOv5s-SE outperforms the baseline YOLOv5s in key metrics such as precision and recall. This improvement validates the effectiveness of the attention mechanism in enhancing the model’s performance. In subsequent research, this method is planned to be deployed on embedded systems to achieve the real-time processing of MMG signals, which will provide more efficient technical support for practical application scenarios such as rehabilitation training.

## 4. Discussion and Conclusions

To address the limitations of traditional MMG signal motion-segmentation methods that rely on manual parameter settings, this study proposes an instance segmentation and classification method for MMG signals based on YOLOv5s-SE, aiming to achieve real-time motion pattern recognition and application using MMG. The specific research content is as follows: First, specialized acquisition equipment was used to systematically collect MMG signals from five upper limb muscles of eight subjects. The collected signals were transformed into image representations in the time domain, and the MMG signals were precisely annotated to construct a dataset named MMG Detection. The dataset contains 1912 images, each containing one or more action signals, covering a total of seven different motion types. Secondly, in this study, the YOLOv5s model was used for training, and on this basis, the SE attention mechanism was introduced for performance optimization. Both models were trained for 100 epochs, and the training results were comprehensively evaluated by multiple indicators.

The results demonstrate that the YOLOv5s-SE model can accurately segment the MMG signals of upper limb movements, and its performance is comparable to that of energy-based methods. At the same time, the model can complete motion pattern recognition synchronously, realizing the real-time classification of actions. The YOLOv5s model achieved an excellent performance of 97.9%, 98.0%, and 99.0% in terms of overall precision, recall, and mAP, respectively. After the introduction of the SE attention mechanism, the YOLOv5s-SE model improved 0.8% and 0.3% in precision and recall, respectively. In addition, the model achieved an accuracy of 98.9% in motion pattern recognition, validating its effectiveness in segmentation and classification tasks. The introduction of the SE significantly improves the model performance, proving the feasibility and research value of the method. In addition to being able to segment and classify individual action signals, the model also performs well in handling consecutive sequences of the same or different motions, which is particularly suitable for application scenarios such as rehabilitation training. Furthermore, the model exhibits good robustness, and the model trained based on one subject’s data can be effectively applied to other subjects’ data. However, further experiments and studies are needed to determine whether the model’s robustness and the corresponding precision, recall, and action recognition performances can meet the requirements if more data from more subjects are added.

In this study, a real-time segmentation and classification method for MMG signals based on the YOLO algorithm is proposed and applied to upper-limb rehabilitation training. The introduction of the SE significantly improves the model’s performance, highlighting the feasibility and research potential of the method. In the future, the method is expected to realize more critical applications in the fields of disease diagnosis, rehabilitation training, and sensory robotic arm control. As artificial intelligence becomes increasingly prevalent in disease prevention and diagnosis within the medical field, there is a growing demand for real-time detection models deployed on embedded systems, necessitating further improvements in model accuracy and speed. Future research may focus on optimizing the training process and backbone network architecture, as well as enhancing model robustness. For example, integrating modules from YOLOv8 or exploring lightweight models and networks could reduce training time and improve detection speed. Additionally, transfer learning techniques and the development of novel network structures are expected to further enhance the robustness and generalization capabilities of the model. These advancements will drive the broader application of MMG signals in rehabilitation and other medical fields.

## Figures and Tables

**Table 1:** Comparison of the overall performance of the two models.

Model	P (%)	R (%)	mAP (%)
YOLOv5s	97.9	98.0	99.0
YOLOv5s-SE	98.7	98.3	99.1

**Table 2:** Model performance statistics analysis table.

Metric	Model	Average Value	Variance	*p*-Value
P	YOLOv5s	97.9%	1.26	0.016 (*p* < 0.05)
	YOLOv5s-SE	98.7%	0.45	
R	YOLOv5s	98.0%	1.40	0.039 (*p* < 0.05)
	YOLOv5s-SE	98.3%	0.72	
mAP	YOLOv5s	99.0%	0.30	0.313 (*p* > 0.05)
	YOLOv5s-SE	99.1%	0.29	

**Table 3:** Performance of the two models on the MMG detection_2 dataset.

Model	P (%)	R (%)	mAP (%)
YOLOv5s	77.3	80.0	88.6
YOLOv5s-SE	83.8	85.8	89.8

## Data Availability

The datasets used in the paper can be found here: [https://doi.org/10.6084/m9.figshare.28690040.v1].
